# Bridging Scales in Flexible Perovskite Solar Cells: Mechanisms, Interfaces, and Applications

**DOI:** 10.1002/advs.202522620

**Published:** 2026-01-22

**Authors:** Yan Wang, Zexin Yu, Chunlei Zhang, Ning Wang, Francesco Vanin, Bo Li, Nan Li, Meng Liao, Zonglong Zhu

**Affiliations:** ^1^ Department of Chemistry City University of Hong Kong Kowloon Hong Kong; ^2^ School of Materials Science and Engineering Central South University Changsha China; ^3^ Department of Materials Science and Engineering City University of Hong Kong Kowloon Hong Kong; ^4^ City University of Hong Kong Shenzhen Research Institute Shenzhen Guangdong China; ^5^ State Key Laboratory of Molecular Engineering of Polymers, Department of Macromolecular Science, and Institute of Fiber Materials and Devices Fudan University Shanghai China

**Keywords:** energy dissipation mechanisms, flexible perovskite solar cells, mechanical flexibility and durability, multi‐scale energy‐based analysis, revolutionary applications

## Abstract

Flexible perovskite solar cells (f‐PSCs) combine an outstanding efficiency‐to‐cost ratio with excellent mechanical properties, offering unique advantages and promising potential in revolutionary applications. Despite systematic advances in device architectures, perovskite regulation, and interfacial‐layer design, the intrinsic correlations among material properties, mechanical behavior, and failure mechanisms remain inadequately investigated. Here, we highlight an energy‐based understanding of recent progress and future prospects of f‐PSCs across microscale perovskite bulk, mesoscale interfacial coupling, and macroscale device/system‐level management. Specifically, the energy dissipation mechanisms in f‐PSCs critically bridge microscopic physicochemical properties and macroscopic material mechanics, which are essential for determining their mechanical durability and operational longevity. Furthermore, this perspective highlights the transformative potential of f‐PSCs in real‐world applications while addressing future advancements in material innovation, interface engineering, and scalable manufacturing techniques to enhance device performance and commercial viability. As research progresses, f‐PSCs are poised to revolutionize the next‐generation emerging photovoltaics, toward a future of higher power conversion efficiency, superior flexibility, and sustainable scalability.

## Graphical abstract

This perspective focuses on the advancement of flexible perovskite solar cells (f‐PSCs) from the energetic point of view, and summarizes the mechanisms, material characteristics, device performance, and failure mechanisms across the microscopic, mesoscopic, and macroscopic scales through mechanical energy dissipation theory. This work provides novel insights for f‐PSC research and highlights the advantages of crosslinking and polymers.

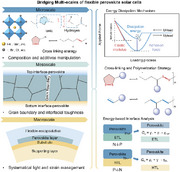

## 1 Introduction

Flexible perovskite solar cells (f‐PSCs) demonstrate unparalleled advantages and irreplaceability in wearable and portable applications due to their excellent efficiency‐cost balance, facile processability, and mechanical reliability [1, 2, 3, 4, 5]. However, compared to rigid perovskite solar cells (r‐PSCs), research on f‐PSCs has still progressed slowly due to unresolved challenges in the field [6, 7]. The reason lies in the fact that f‐PSCs face more unaddressed complexities compared to their rigid counterpart, benefiting from a mature understanding and detailed investigation of atomic‐to‐molecular‐scale functionalities [8] and microstructural construction [9, 10, 11, 12]. Therefore, a systematic research framework is lacking to bridge microscopic physicochemical properties and macroscopic material mechanics through a mesoscopic energy‐based perspective.

Such an energy‐based approach can provide feasible and comprehensive insights into the mechanical behavior of f‐PSC across multiple dimensions. From the microscopic scale, research on flexible devices can take inspiration from the strategies employed in high‐efficiency r‐PSCs, such as manipulating chemical compositions [13, 14] and tuning intermolecular interactions [15]. These strategies enhance the intrinsic mechanical properties of bulk perovskite materials [16]. On the macroscopic level, f‐PSCs can benefit from the fabrication and optimization experience of rigid perovskite single‐junction and tandem modules, [17, 18, 19] and mature testing methodologies and theoretical investigations established in metals and polymer fields at the macroscale [20, 21, 22, 23, 24]. However, they still face major challenges such as high cost [25] and more complex failure mechanisms [26, 27]. Therefore, the key to bridging scales in a multiscale framework is to focus on an energy‐based mechanical analysis, including grain boundary (GB) interactions, [28, 29] interfacial fracture energy, [30, 31] and energy dissipation mechanisms, [23, 32, 33, 34] to provide critical insights into the mechanical behavior and reliability of f‐PSCs.

In this perspective, we provide a summary and highlight the progress in f‐PSC research from an energy‐based approach [23, 27, 35]. We evaluate the mechanical properties of f‐PSCs, focusing on building strong interconnections across three dimensions: the microscale (bulk perovskite), the mesoscale (interfacial connections), and the macroscale (systemic management). Furthermore, we highlight the vital application areas of f‐PSCs, [36] including building‐attached photovoltaics (BAPV) and building‐integrated photovoltaics (BIPV), [37, 38] wearable and portable devices, [3] and aerospace applications [10]. Looking ahead, breakthroughs in perovskite materials, interface engineering, and scalable manufacturing technologies will be critical for improving device performance and commercial feasibility [39]. As technology continues to advance, f‐PSCs are poised to drive the photovoltaic industry toward a future of higher efficiency, greater flexibility, and enhanced sustainability, bringing revolutionary changes to the field of clean energy.

## 2 Comprehensive Multiscale Understanding of Flexible Perovskite Solar Cells

This section provides a systematic overview of flexible solar cells, focusing on their device performance, materials properties, and energetic mechanism at the beginning [40]. Then an in‐depth insight into the fracture mechanisms and mechanical toughness regulation strategies of f‐PSCs across different scales [14, 35, 41]. By integrating energy‐based principles, we aim to unravel the complex interplay among material properties, interfacial linking, device management, and external stresses, thereby providing guidance for the development of robust and high‐ performance f‐PSCs tailored for practical applications.

### 2.1 Efficiency and Mechanical Performance of Flexible Solar Cells

**FIGURE 1:**
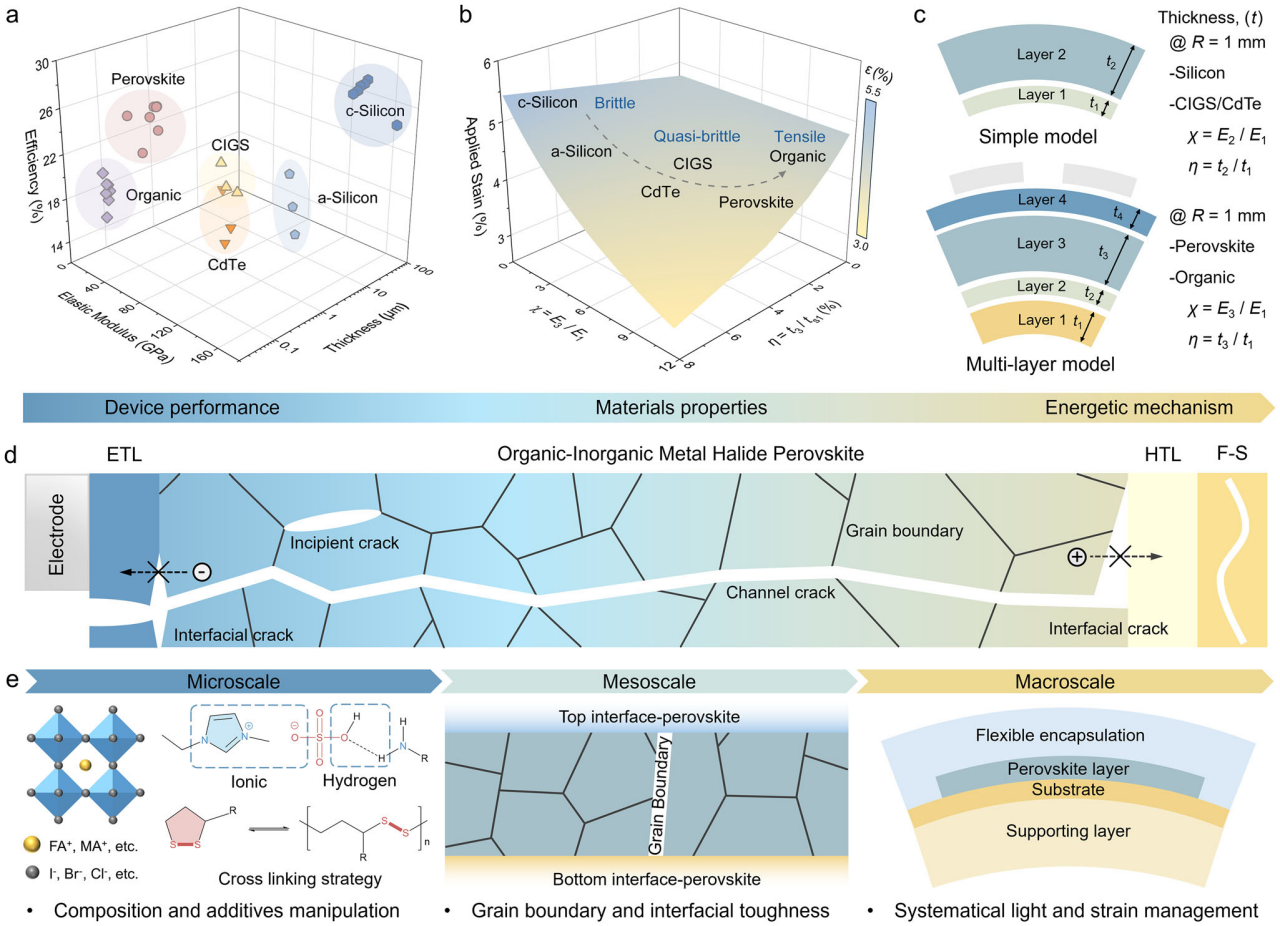
Introduction to flexible perovskite solar cells (f‐PSCs) and the fracture mechanism. a) Efficiency versus elastic modulus and thickness of active layers of different flexible solar cells (f‐SCs). b) Applied strain at bending radius (1 mm) of corresponding f‐SCs versus *χ* and *η* parameters. c) The illustration and definition of *χ* and *η* for the simple model and multi‐layer model of various f‐SCs, respectively. d) The fracture mechanism and performance failure of flexible perovskite film through (1) incipient crack, (2) channel crack, and (3) interfacial crack with the increasing of *σ*_appl._ . e) The critical understanding of flexible perovskite solar cells (f‐PSCs) under three different dimensions from an energetic perspective.

F‐PSCs have emerged as a leading technology in the field of flexible photovoltaics, achieving a certified power conversion efficiency (PCE) exceeding 23% while exhibiting exceptional mechanical properties [18, 42, 43, 44, 45] This is attributed to their low elastic modulus [30, 35] (∼10–20 GPa) and ultrathin active layer thickness (less than 1 µm), as illustrated in Figure 1a. In contrast, crystalline silicon (c‐Si) solar cells require specialized structural designs and a reduction in absorber layer thickness to achieve limited flexibility [46, 47]. Although other inorganic thin‐film photovoltaic technologies, such as amorphous silicon (a‐Si), [48, 49] copper indium gallium selenide (CIGS), [50, 51] and cadmium telluride (CdTe), [52, 53, 54] can be fabricated at micrometer scale thicknesses, their performance‐to‐cost ratios and mechanical properties fall short of those of f‐PSCs [5, 55]. Meanwhile, flexible organic photovoltaic devices (f‐OPVs) are hindered by lower efficiency, poor stability, and prohibitively high material costs [56, 57].

Unlike PCE, which is a well‐defined photovoltaic metric, the mechanical performance of flexible solar cells lacks unified and widely accepted evaluation standards [58]. This is primarily due to the subjective selection of testing methods and variations in laboratory equipment for measurement. While a recent study has called for standardized testing protocols for f‐PSCs, [58] no ISOS‐based flexible bending standards have been established yet [59]. It is recommended to evaluate the mechanical stability by a cyclic bending test over 1000 cycles at 1% applied strain. In this perspective, we employ a levelized computational analysis to quantify the applied strain induced in various flexible photovoltaic devices under a bending radius (*R*) of 1 mm, which is considered suitable for meeting the requirements of most practical applications, [35] as shown in Figure 1b. For materials with relatively simple structures, such as Si, CIGS, and CdTe, strain calculations can be performed using a conventional simple model (given by Equation (1)).

For emerging thin‐film flexible devices, such as perovskites and OPVs, more precise multilayer structural model calculations are required (given by Equation (2)). Key parameters in these calculations include the elastic modulus ratio (*χ*) and the thickness ratio (*η*), [60] where:

(1) $$\chi_{\mathrm{Type}1}=\frac{E_{2}}{E_{1}};\eta_{\mathrm{Type}1}=\frac{t_{2}}{t_{1}}$$

(2) $$\chi_{\mathrm{Type}2}=\frac{E_{3}}{E_{1}};\eta_{\mathrm{Type}2}=\frac{t_{3}}{t_{1}}$$

Here, *E*_i_ and *t*_i_ represent the elastic modulus and thickness of each layer respectively, as shown in Figure 1c.

As demonstrated in Figure 1b, the computational results reveal that under identical external stress conditions (bending herein), perovskite materials exhibit the lowest applied strain, indicating their significant advantage in mechanical resilience [55, 61]. The specific computational details are further elaborated in Section 5, focusing on strain management.

While the excellent mechanical properties of f‐PSCs underscore their potential for flexible applications, [26] understanding their failure mechanisms under external stresses is crucial for ensuring long‐term stability. The fracture behavior of f‐PSCs, especially under bending and strain, directly affects their operational durability [62, 63, 64, 65]. In the following section, we explore the fracture mechanisms and manipulation strategies of f‐PSCs, focusing on how energy dissipation across multiple scales can mitigate mechanical degradation and enhance reliability.

### 2.2 Fracture Mechanism and Manipulation Principles for Mechanical Stability

The mechanical stability and performance loss of f‐PSCs primarily suffer from structural and material collapse caused by cracking and delamination of the active layer under high‐strain conditions [65]. As shown in Figure 1d, the polycrystalline perovskite films are prone to stress concentration at GBs during bending. Consequently, the incipient crack typically occurs when the film bends toward the flexible substrate. This primary behavior is determined by the bulk properties of perovskite, such as the elastic modulus, intrinsic critical fracture energy (*G*_c_), residual stress (*σ*_r_), and the length of the GB [35, 66]. Notably, *σ*_r_ can evolve under high applied stress (*σ*_appl._) and thermal cycling, and the capability of the perovskite film to release strain (i.e., stress dissipation/relaxation) critically modulates the local stress concentration at defect‐rich GBs [67]. As the *σ*_appl._ increases, these microfractures propagate along the thickness direction, ultimately forming a channel crack from the top surface to the bottom interface of the device. This process is dependent on the intergranular and interfacial cohesion, density and distribution of GB, and film thickness. The fracture mechanism is also environment‐sensitive with humidity and temperature, which might affect the interfacial cohesion, plastic relaxation and accelerate ion migration, leading to stress accumulation, stress release, and crack growth, especially under complicated environmental stresses including bending, heat, moisture, illumination and bias. Continued or increased stress accumulation eventually leads to interfacial cracking, severely compromising device performance and stability [45, 62, 63, 65]. This behavior is determined by complicated material properties, including interfacial roughness, modulus and thermal expansion coefficient matching, and strain dissipation capacity, which together govern both stress concentration and stress release at the interfaces [22, 34, 68]. Therefore, to elucidate the fracture failure mechanisms of f‐PSCs, particularly to suppress interfacial delamination, it is essential to bridge the micro‐, meso‐, and macro‐scales through an energetic investigation as depicted in Figure 1e.

At the microscale, the composition of perovskites determines their bulk *E* and *G*_c_, while additive engineering can effectively reduce the fracture driving forces by regulating σr and grain size [42, 62, 69]. Additionally, through crosslinking and polymerization strategies, introducing additional intermolecular interactions enhances the effective fracture toughness (*K*_Ic_) of the material via mechanical energy dissipation [23, 34, 56, 64]. At the mesoscale, the interaction strength between grains and at various interfaces is critical to mechanical stability. The introduction of multi‐anchoring, functional self‐healing structures [70] can mitigate the mechanical degradation of f‐PSCs by healing cracks and suppressing crack propagation [28, 29, 61]. At the macroscopic scale, achieving an efficient and reliable flexible perovskite integrated system necessitates the management of light utilization and strain dispersion [4, 56, 71]. Bridging these three scales highlights the design and optimization of f‐PSCs from an energy perspective, paving the way for their widespread deployment.

## 3 Bulk Toughening Engineering of Flexible Perovskites: Microscale

Intrinsic bulk perovskite properties, such as ion types, crystal parameters, and coordination states, fundamentally determine their mechanical and optoelectronic performance at the nanoscale [13, 72, 73, 74]. This section discusses the bulk toughening strategies, including compositional and additive engineering, hybrid structural designs, especially crosslinking and polymerization that facilitate energy dissipation, to enhance mechanical strength and resistance to cracking and delamination under $\sigma_{\mathrm{appl}.}$.

### 3.1 Compositional, Additive, and Hybrid‐Structural Manipulations

**FIGURE 2:**
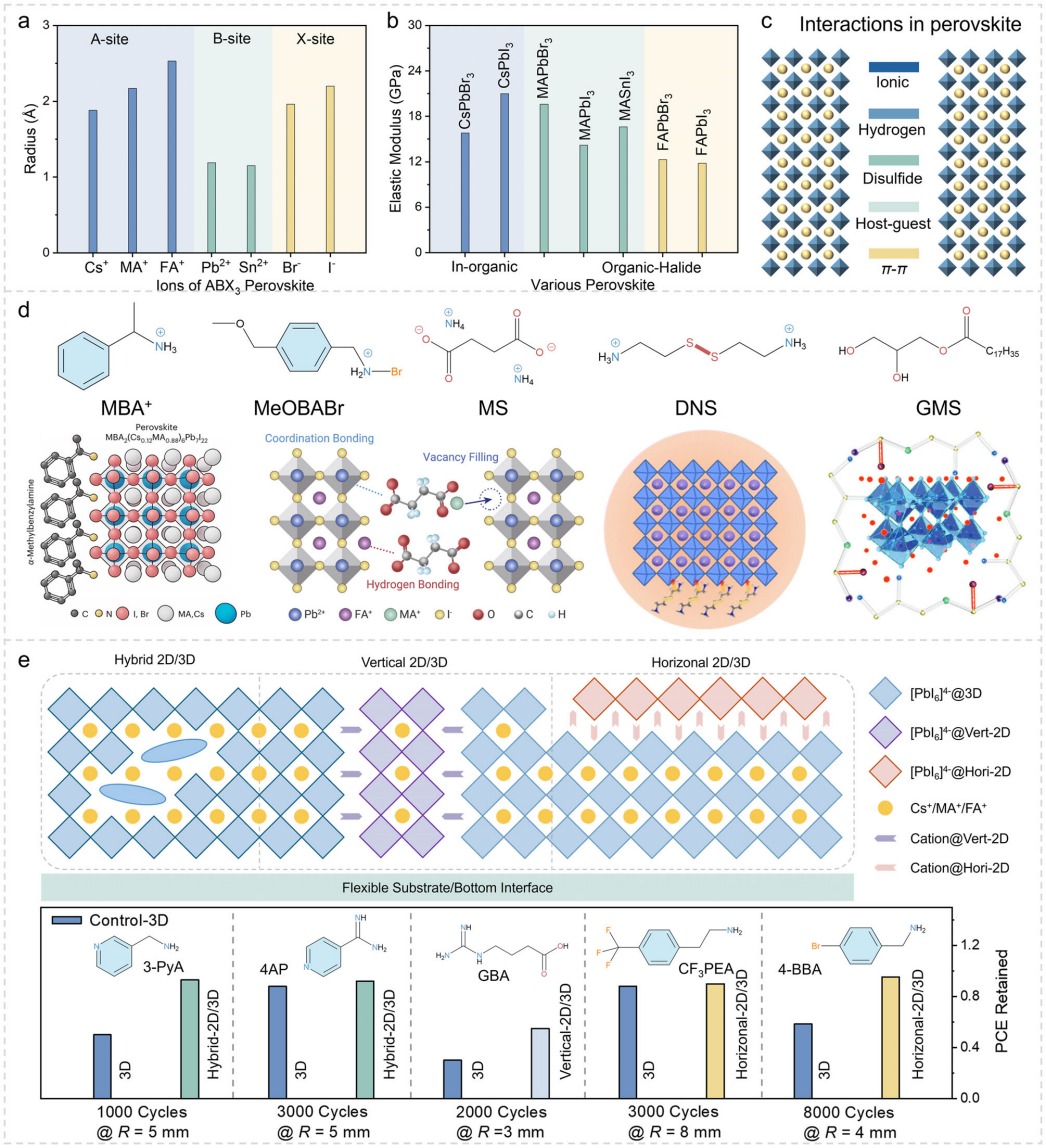
Composition, additive, and structure engineering for f‐PSCs. a) Ionic radii of different cations and anions of ABX_3_‐type perovskites. b) Elastic moduli of different compositions of perovskite. c) Various interactions in the bulk perovskites. d) Molecular structures of effective additives for f‐PSCs and the corresponding microscale interactions with perovskite. Reproduced with permission [10]. Copyright 2024, Springer Nature. Reproduced with permission [81]. Copyright 2022, Elsevier. Reproduced with permission [80]. Copyright 2024, RSC Publishing. Reproduced with permission [82]. Copyright 2024, Wiley‐VCH. e) The illustration of hybrid, vertical, and horizontal 2D/3D heterostructures of perovskites and corresponding bending stabilities compared with 3D perovskite as counterparts, respectively.

Different combinations of A‐site, B‐site, and X‐site ions with various ionic radii and polarizability significantly affect the bulk elasticity and crystal toughness of perovskites, [13, 72, 75, 76] as summarized in Figure 2a,b. A‐site organic cations (e.g., methylammonium cation, MA^+^, and formamidinium cation, FA^+^), owing to their relatively larger effective ionic radii and higher polarizability, typically lead to corresponding perovskites with lower elastic moduli [13, 75]. Accordingly, tailoring the A‐site cation composition has been demonstrated to effectively regulate the mechanical properties of perovskite materials. Meanwhile, mixed‐cation (e.g., Cs^+^/MA^+^/FA^+^) or mixed‐halide (e.g., Br^−^/I^−^) formulations can yield more complex effects, which are often strongly dependent on thin‐film processing conditions and the fabrication environment [77]. However, the compositional engineering may introduce non‐negligible side effects, such as bandgap shifts and phase segregation, which can complicate the simultaneous optimization of mechanical robustness and optoelectronic performance. Consequently, it is essential to balance photovoltaic performance and mechanical properties to achieve superior efficiency while maintaining excellent mechanical robustness.

The addition of functional molecules can also improve both the mechanical and photovoltaic properties of perovskites [4, 38, 78, 79] by introducing diverse interactions as illustrated in Figure 2c. For instance, 4‐(methoxy)benzylamine hydrobromide (MeOBABr) modification (as shown in Figure 2d) has been shown to effectively promote perovskite crystallization and reduce *σ*_r_, thereby lowering the surface stress of perovskites from 25 to 12.1 MPa [42]. The use of multifunctional small molecule ligands, which can coordinate perovskites, can similarly toughen perovskite grains [80, 81, 82]. The additives may suffer from issues such as additive migration, sensitivity to precursor composition and processing window, and potential long‐term instability or electronic side effects.

Constructing low‐dimensional/3D (LD/3D) perovskite heterostructures is also effective in improving the bending behavior of f‐PSCs [83, 84, 85]. For example, in the case of 2D/3D perovskite heterostructures, there are three typical mixing methods, as illustrated in Figure 2e. 3D perovskites randomly dispersed within the 3D perovskite bulk phase (hybrid 2D/3D); [83, 86] 2D perovskites vertically embedded in 3D perovskite grains (Hori‐2D/3D); [87, 88, 89, 90, 91] and 2D perovskite layers covering the surface of 3D perovskites (Vert‐2D/3D) [69, 90, 92]. Although the three 2D/3D heterostructures have been reported to improve both the PCE and the bending stability of f‐PSCs, the Vert‐2D/3D heterostructure is supposed to exhibit superior flexibility through multilayer stress dispersion as illustrated in Figure 2e [2, 22, 93]. Beyond such structural stress‐management strategies enabled by LD/3D heterostructures, energetic toughening can be achieved by introducing polymerization‐ and cross‐linking–derived dissipation pathways, which is the focus of the following section [33].

### 3.2 Energetic Toughening Strategies Achieved by Cross‐Linking and Polymerization

Cross‐linking and polymerization offer energetic toughening routes for perovskite films by introducing dissipation mechanisms such as dynamic reversible bonding, molecular‐chain slippage, and network‐like constraints, thereby bridging microscale (bonding/defect) and mesoscale (GB/interface) scales [94, 95, 96]. These mechanisms can mitigate stress concentration and retard crack initiation and propagation under repeated bending.

**FIGURE 3:**
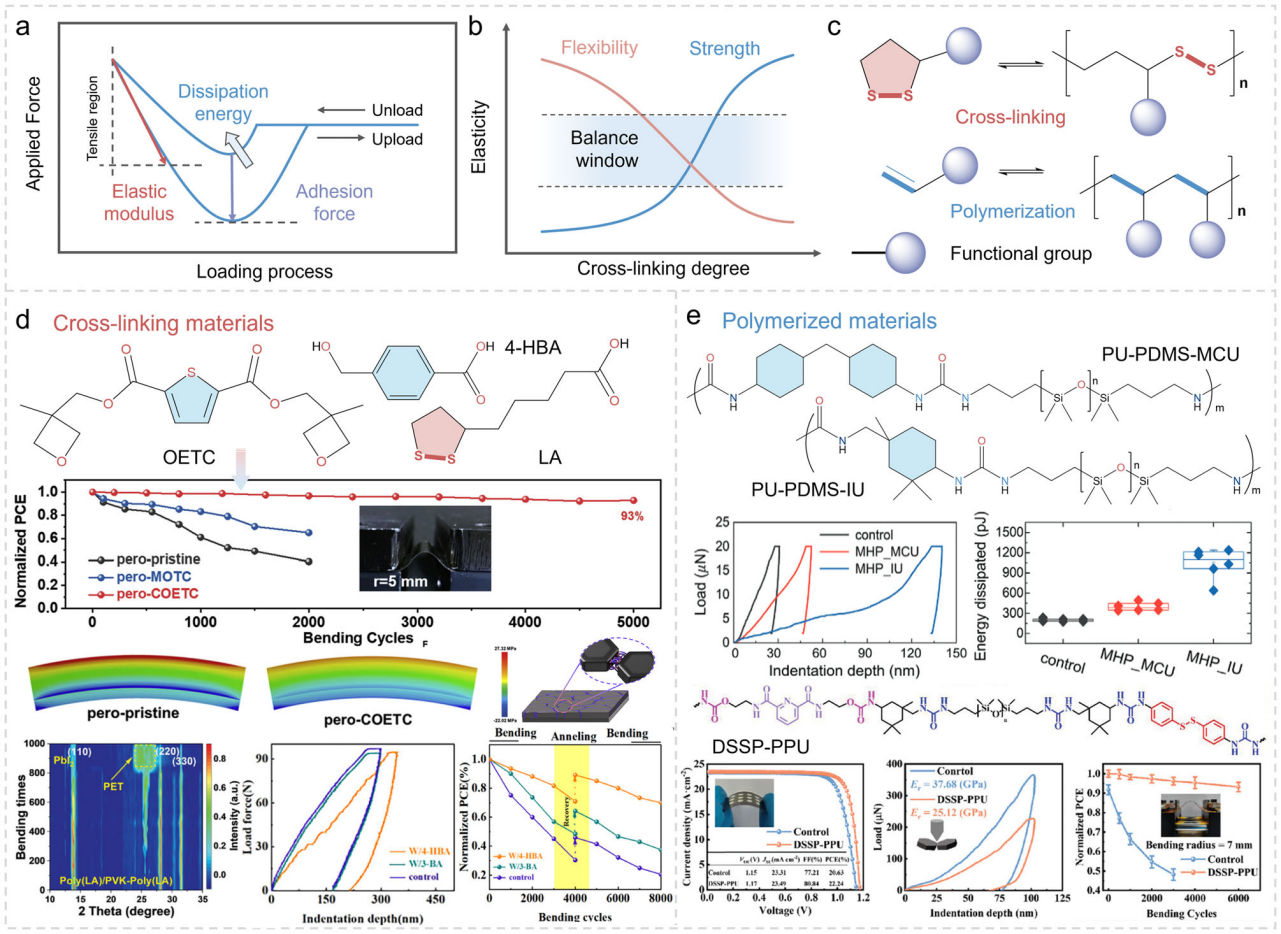
Strengthening perovskite through energy dissipation mechanism by cross‐linking and polymerization methods. a) Illustration of elastic formation, adhesion force, and dissipation energy during upload and unload of samples through DMT modulus fitting. b) The balance between flexibility and strength of materials under various cross‐linking degrees. c) Energy dissipation strategies through cross‐linking and polymerization. d) The molecular structure and corresponding investigations of OETC, LA, and 4‐HBA as cross‐linking agents for f‐PSCs. Reproduced with permission [101]. Copyright 2023, Elsevier. Reproduced with permission [63]. Copyright 2023, Wiley‐VCH. Reproduced with permission [103]. Copyright 2025, Wiley‐VCH. e) The molecular structure and corresponding investigations of PU‐PDMS‐MCU, PU‐PDMS‐IU, and DSSP‐PPU as polymerized materials for f‐PSCs. Reproduced with permission [105] Copyright 2022, Wiley‐VCH. Reproduced with permission [106]. Copyright 2023, Wiley‐VCH.

From an energetic perspective, such toughening is often manifested as an increased mechanical dissipation energy (*w*_D_). As shown in Figure 3a, *w*_D_ is quantified by the area enclosed by the loading–unloading hysteresis loop during testing [23, 34, 97, 98]. This dynamic dissipation process depends on both the elastic response and interfacial interactions (e.g., adhesion and reversible bonding), which can be described by Equations ((3), (4), (5)).

(3) $$G_{D}\approx 2\alpha \bar{s}\left(\bar{\lambda }-1\right)h$$

where *α* represents stress–strain hysteresis, $\bar{s}$ and $\bar{\lambda }$ represent the average stress and strain, and *h* represents the substantial size.

(4) $$\alpha =\frac{w_{D}}{\bar{s}\left(\bar{\lambda }-1\right)}$$

where *w*_D_ can be given by

(5) $$w_{D}=\sum_{i=1}^{3}\oint s_{i}d\lambda_{i}$$

where *λ_i_* are the principal stretches in three directions, *s_i_* the corresponding principal stresses, and ∮ represents integration over the hysteresis loop. To quantitatively evaluate energy dissipation in f‐PSCs, Atomic Force Microscopy (AFM) force‐distance measurement, instrumented nanoindentation, and cyclic tensile or bending experiments can be used. At the nanoscale, PeakForce Quantitative Nanomechanical Mapping (PFQNM)‐AFM can provide spatially resolved dissipation using the Derjaguin‐Muller‐Toporov (DMT) contact model to extract an apparent elastic modulus, and the hysteresis between the approach–retract branches for local mechanical dissipation, enabling comparison between grains and GBs. However, because DMT is most reliable under weak‐adhesion, near‐elastic contact with relatively small contact radii, systems with strong adhesion and pronounced plastic responses (e.g., polymer‐rich functional materials) should be cross‐checked using alternative contact models (e.g., Johnson‐Kendall‐Roberts, JKR model) and complementary mechanical tests.

Nevertheless, as illustrated in Figure 3b, flexibility and mechanical strength are typically mutually exclusive [56, 99]. Therefore, controlling the degree of crosslinking and polymerization is crucial (as shown in Figure 3c) to achieve a balance between these two factors, which lacks investigation in f‐PSCs [100]. For instance, cross‐linking monomers such as bis((3‐methyloxetan 3‐yl) methyl) thiophene‐2,5‐dicarboxylate (OETC), [101] lipoic acid (LA), [102] and 4‐hydroxybenzoic acid (4‐HBA) [103] have been used within f‐PSCs (Figure 3d), or elastic polymers such as [27, 104] PU‐PDMS‐MCU, PU‐PDMS‐IU, [105] and DSSP‐PPU [106] (Figure 3e) have been introduced to construct effective mechanical energy dissipation pathways [34, 107]. These materials significantly expand the dynamic and reversible cross‐linking domains and show great potential as energy‐dissipating materials for toughening perovskite active layers [22, 31, 33]. By integrating these multiscale mechanisms, crosslinking and polymerization provide a robust framework for improving the mechanical stability of f‐PSCs while maintaining their optoelectronic performance.

## 4 Perovskite Interface Linking Materials for Tailored Functionalities: Mesoscale

Interfacial stability is vital for ensuring mechanical durability and operational reliability at the mesoscale. This section explores strategies for designing interface materials with tailored functionalities, focusing on enhancing adhesion, mechanical resilience, and long‐term stability to address the unique challenges of f‐PSCs [59, 108].

### 4.1 Bottom Interface Materials Based on Adhesive Properties

**FIGURE 4:**
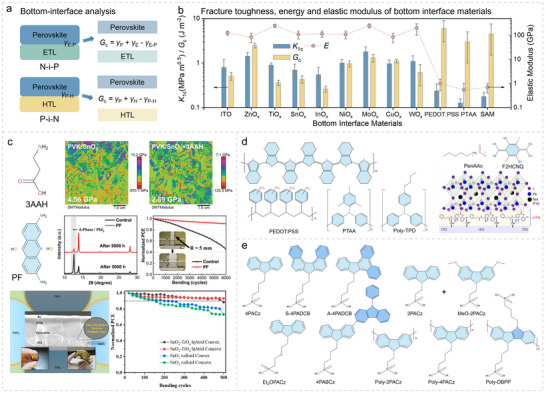
Bottom interfacial materials properties and mechanical analysis. a) Illustration of bottom‐interface cracking analysis for N‐i‐P and P‐i‐N f‐PSCs. b) Summarized mechanical properties (*K*_Ic_, *G*_c_, and *E*) of different bottom interface materials. c) Molecular structure and investigations of 3AAH, PF and SnO_2_/TiO_2_ hybrid nanoparticles. Reproduced with permission [111]. Copyright 2023, Wiley‐VCH. Reproduced with permission [43]. Copyright 2024, Wiley‐VCH. Reproduced with permission [112]. Copyright 2022, American Chemical Society. d) Molecular structure of PEDOT:PSS, PTAA, Poly‐TPD, PenAAc, F2HCNQ, and ET. Reproduced with permission [18]. Copyright 2024, Springer Nature. e) Chemical structures of various small‐molecular and polymerized SAMs used in f‐PSCs.

The bottom interfaces play pivotal roles in mitigating crack propagation and delamination while bending. To mitigate this issue, it is essential to construct bottom interface materials (BIMs) with stronger adhesion properties, as evaluated in Figure 4a. Herein, Figure 4b summarizes the *K*_Ic_, *G*_c_, and *E* of commonly used BIMs, [109] while the organic BIMs exhibit an impressive advantage in terms of *G*_c_. However, the scarcity of *n*‐type organic BIMs suggests the potential advantages of *p‐i‐n* structures for f‐PSCs [110].

Treating metal oxide layers in *n‐i‐p* devices with organic materials is an effective strategy (as shown in Figure 4c). For instance, surface modifications of tin dioxide (SnO_2_) using 3‐aminopropionic acid hydroiodide (3AAH) [111] and proline hydrochloride (PF) [43] can effectively reduce the overall *E*, thereby improving mechanical stability. Additionally, mixing metal oxides (e.g., SnO_2_‐Titanium dioxide (TiO_2_)) hybrid nanoparticles can effectively mitigate delamination under bending stress, [112] as illustrated in Figure 4d.

For *p‐i‐n* architectures, although polymeric hole transport layers (HTLs) such as poly[bis(4 phenyl)(2,4,6‐trimethylphenyl)amine] (PTAA) [113], poly(N,N'‐bis‐4‐butylphenyl‐N,N'‐bisphenyl)benzidine (poly‐TPD) [114], and poly(3,4‐ethylenedioxythiophene) poly(styrene‐sulfonate) (PEDOT:PSS) [115] typically exhibit excellent mechanical properties as shown in Figure 4e, their optoelectronic performance is inferior to carbazole‐based phosphate derivatives‐based self‐assembled monolayers (SAM)‐based HTLs, [116, 117, 118] limiting their ability to achieve higher PCE of f‐PSCs [108]. On the other hand, the high roughness, low conductivity, and chemical sensitivity of flexible substrates impose stringent requirements on the processing of BIMs and perovskite films. Organic molecules such as pentylammonium acetate (PenAAc), [45] 3,6‐difluoro‐2,5,7,7,8,8‐hexacyanoquinodimethane (F2HCNQ), and entinostat (ET) [18] can partially address these challenges (as shown in Figure 4e). Furthermore, structural engineering of SAM molecules together with process optimization represents a more promising solution [44, 119, 120]. For example, introducing an oxygen atom into the linker segment (e.g., Et_2_OPACz [121]) or extending the π‐conjugated plane of the terminal group (e.g., A‐4PADCB and 4PABCz [122]) can improve both the device performance and mechanical stability. The mixed SAMs combined with 2PACz and MeO‐2PACz achieved an excellent PCE over 25% of flexible perovskite tandem solar cells. Specifically, considering the intrinsic mechanical characteristics of polymers, polymerized SAMs (like poly‐2PACz, [123, 124] poly‐4PACz, [125] poly‐DBPP, [126] etc.) are expected to be highly promising for flexible perovskite applications, although this will require more in‐depth investigations. The molecular structures discussed above are summarized in Figure 4f [123, 127].

### 4.2 Top Interface Materials for Suppressing the Degradation

**FIGURE 5:**
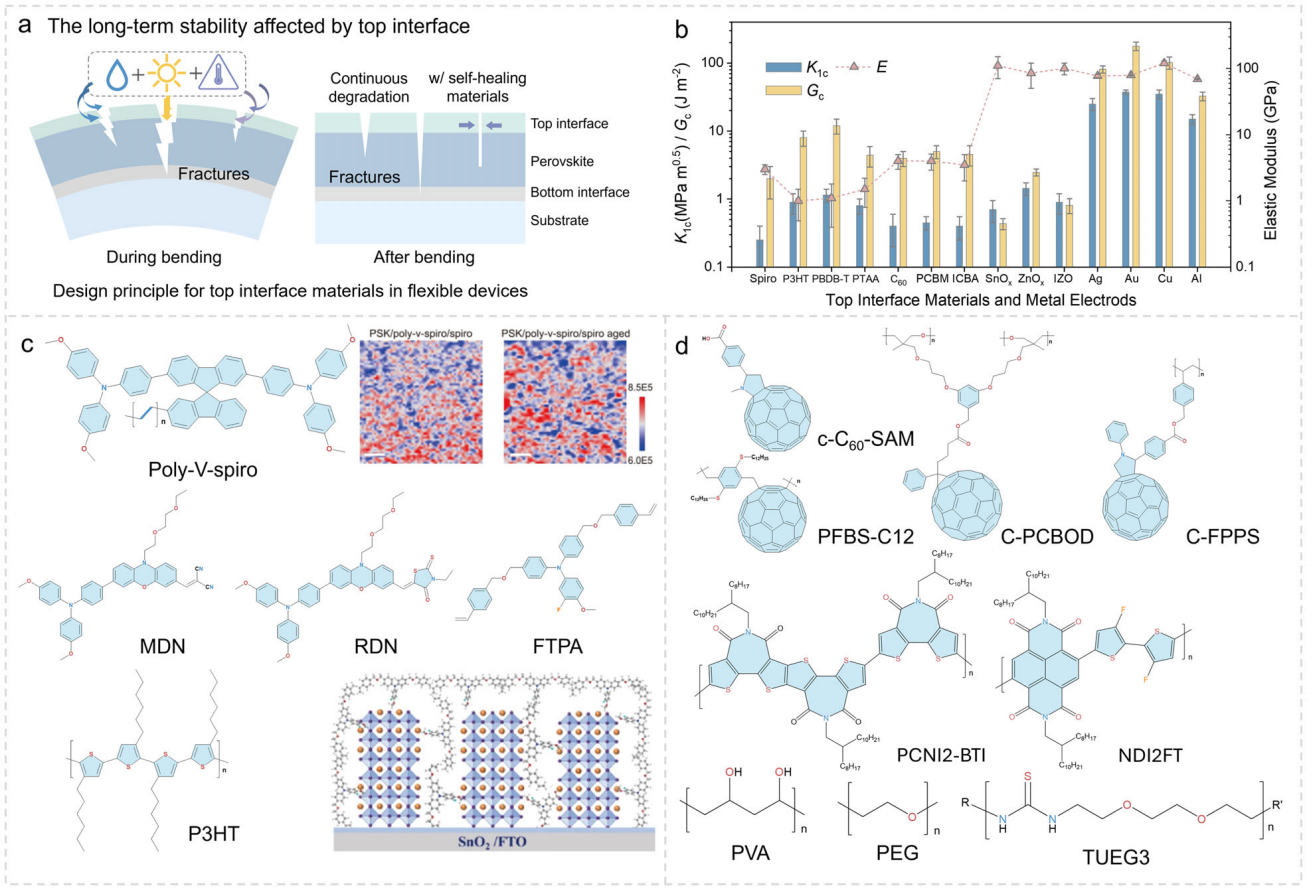
Top interfacial materials for suppressing the perovskite degradation. a) Long‐term stability influenced by the top interface to suppress perovskite degradation under humid, light, and chemical conditions. b) Summarized mechanical properties (*K*_Ic_, *G*_c_, and *E*) of different top interface materials and metal electrodes. c) Molecular structure and stability test of poly‐V spiro. Reproduced with permission [131] Copyright 2024, Wiley‐VCH. d) Molecular structure and investigations of MDN, RDN, FTPA, and P3HT. Reproduced with permission [132]. Copyright 2022, Springer Nature. e) Chemical structure of c‐C_60_‐SAM, PFBS‐C12, C‐PCBOD, C‐FPPS, PCNI2‐BTI, NDI2FT, PVA, PEG, and TUEG3, respectively.

The properties of the top interfacial materials (TIMs) significantly influence the long‐term stability of f‐PSCs in real‐world application scenarios [41, 59]. Although the TIMs in f‐PSCs are typically similar to those in rigid counterparts, the TIMs are prone to prior channel cracking under bending stress [41]. Therefore, an in‐depth investigation into the mechanical behavior of TIMs remains essential. As shown in Figure 5a, the presence of top cracks exposes the perovskite layer to external environmental conditions, accelerating device degradation and failure under external stress. Figure 5b summarizes *K*_Ic_, *G*_c_, and *E* parameters for commonly used TIM materials and metal electrodes as well [128, 129].

Compared to other HTL materials, 2,2′,7,7′‐tetrakis[N, N‐di(4‐methoxyphenyl)amino]‐9,9′ spirobifluorene (Spiro‐OMeTAD) exhibits poor resistance to mechanical deformation and is highly susceptible to oxidative degradation [71]. As a result, Spiro‐based HTLs often face significant stability challenges under cycled bending conditions [130]. The polymerization of Spiro (poly‐V‐Spiro, as shown in Figure 5c) has been proven to surpass some of these issues while maintaining decent hole transport properties [131]. In contrast, polymeric HTLs such as P3HT exhibit better mechanical resilience and water/oxygen barrier properties compared with small molecular‐based BIMs as shown in Figure 5d [132]. However, they still suffer from low hole transport efficiency and energy level mismatches, which limit their overall performance [133].

For *p‐i‐n* architectures, fullerenes and their derivatives are widely used as electron transport layers (ETLs) [134, 135] To mitigate the problem of excessive aggregation and structural transformation, cross‐linking [136, 137] and polymerization strategies [138] have also been employed in this context, as shown in Figure 5e. Recently, non‐fullerene polymer ETLs such as PCN2‐BTI [139] and NDI2FT [140] (Figure 5e) have emerged as promising alternatives compared with C_60_ and PC_61_BM, offering improved morphological robustness and suppressed aggregation and phase instability under light, thermal stresses. More importantly, their polymeric properties can provide a better energy dissipation pathway with high adhesion force, enabling more uniform, crack‐tolerant ETLs while maintaining efficient electron extraction during mechanical measurements and protecting the perovskite layer from moisture.

Another alternative approach is to introduce self‐healing polymers as TIMs to tensile the f‐PSCs, such as PVA, [141] PEG, [142] and TUEG3 [143]. These materials can promote the elimination and mitigation of channel cracks as the f‐PSC recovers to its original planar state, significantly enhancing the long‐term stability of f‐PSCs and suppressing performance degradation. However, the cost and accessibility of these functional materials still greatly limit the development and commercialization of f‐PSCs.

## 5 Light and Strain Management of Flexible Perovskite Solar Cells: Macroscale

**FIGURE 6:**
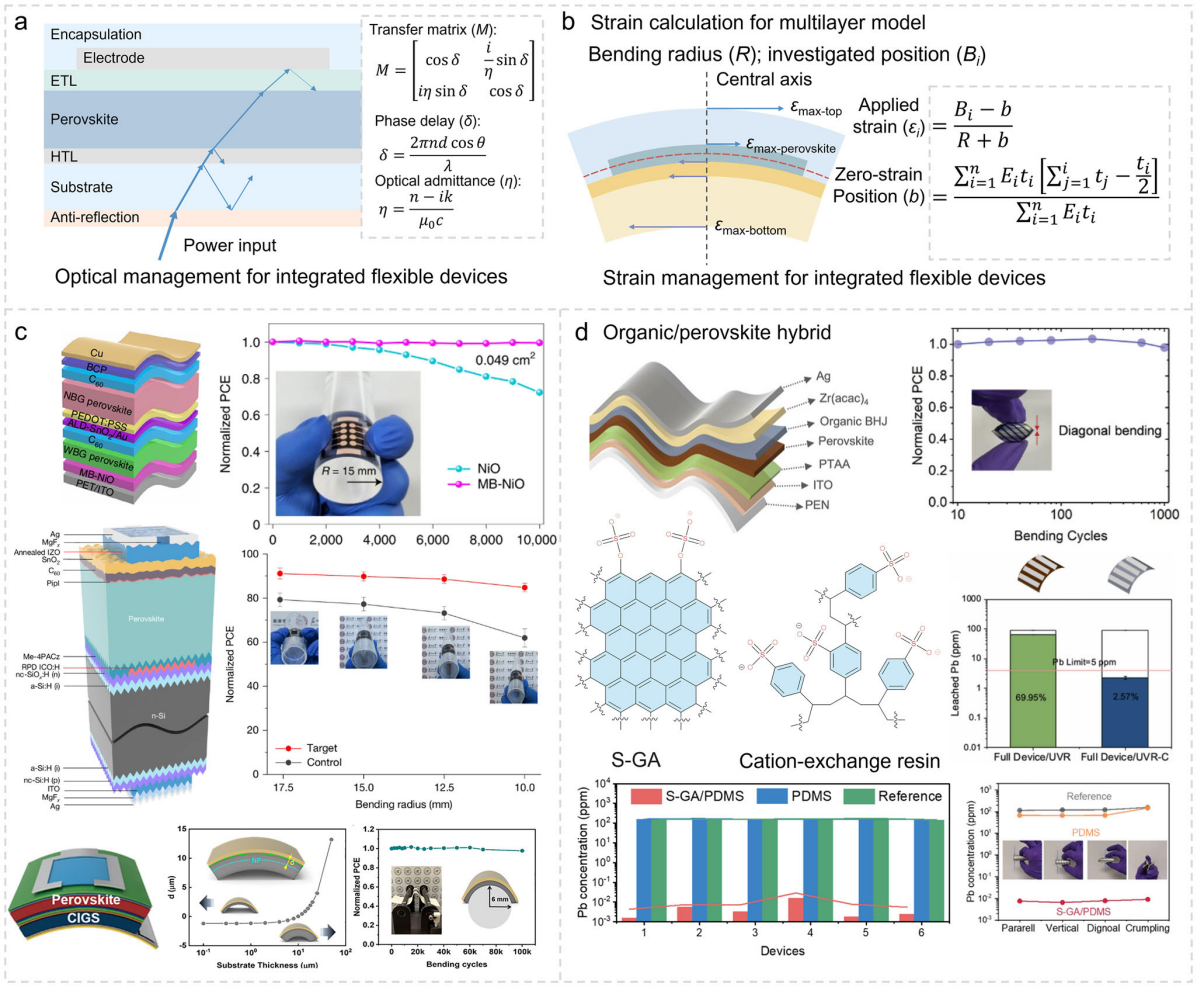
Optical and strain management for integrated flexible perovskite systems. a) Optical management analysis of f‐PSCs by calculating the transfer matrix of each layer. b) Strain management analysis of f‐PSCs by calculating the applied strain of each layer through a multi‐layer model. c) Device structure and corresponding mechanical stability of flexible all‐perovskite tandem solar cells, flexible silicon‐perovskite tandem solar cells, and flexible CIGS‐perovskite tandem solar cells, respectively. Reproduced with permission [119]. Copyright 2022, Springer Nature. Reproduced with permission [149]. Copyright 2025, Springer Nature. Reproduced with permission [145] Copyright 2025, Cell Press. d) Organic‐perovskite hybrid solar cells. Reproduced with permission [150]. Copyright 2021, Wiley‐VCH. e) Flexible encapsulation materials (S‐GA and cation‐exchange resin) and corresponding applications. Reproduced with permission [152] Copyright 2021, Wiley‐VCH. Reproduced with permission [153]. Copyright 2021, Elsevier.

Building on the advancements in bulk perovskite phase and interface linking for f‐PSCs, optimizing optical and mechanical performance at the macroscopic scale is essential for real‐ world applications. This section explores photon harvesting and stress dissipation approaches, integrating light management (Figure 6a) and strain management (Figure 6b) to maximize efficiency and durability in f‐PSCs [30, 74].

### 5.1 Light Management for Improving Photon Harvesting

The flexible substrates, like polyethylene terephthalate (PET) and polyethylene naphthalate (PEN), compromise on transparency and surface smoothness in order to achieve excellent mechanical flexibility. Therefore, effective light management is necessary to compensate for the energy losses caused by these limitations. For the multilayer structure, light utilization follows the equations provided below (Equations ((6), (7), (8))). through the transfer‐matrix method.

(6) $$M=\left[\begin{array}{cc} \cos\delta & \frac{i}{\eta_{M}}\sin\delta \\ i\eta \sin\delta & \cos\delta \end{array}\right]$$

where *M* is the transfer matrix, *δ* represents phase delay:

(7) $$\delta =\frac{2\pi nd\cos\theta }{\lambda }$$

and *η*_M_ represent the optical admittance:

(8) $$\eta_{M}=\frac{n-ik}{\mu_{0}c}$$

Therefore, the thickness of the interfacial layer and active layer, as well as the introduction and modulation of the antireflection layer, should be systematically evaluated through this method before being comprehensively optimized. Meanwhile, as for flexible tandem devices, this aspect also requires reevaluation rather than directly applying the experience from rigid tandem devices, as shown in Figure 6c for flexible all‐perovskite, [119] perovskite/silicon, [144] and perovskite/CIGS tandem devices [145].

### 5.2 Strain Management for Boosting Robustness and Reliability

For multilayer structures during the bending process, the precise stress evaluation formula satisfies the relationship described by Equations (9) and (10) [20, 60, 146]:

(9) $$b=\frac{\sum_{i=1}^{n}E_{i}t_{i}\left[\sum_{j=1}^{i}t_{j}-\frac{t_{i}}{2}\right]}{\sum_{i=1}^{n}E_{i}t_{i}}$$

where *b* represents the zero‐strain position, and the applied strain *ε_i_* is defined as

(10) $$\varepsilon_{i}=\frac{B-b}{R+b}$$

where B is the position of the investigated position.

The as‐calculated b parameter corresponds to the neutral plane, where the stress is zero under a specific bending radius for flexible devices. This neutral plane should ideally be located within the perovskite active layer to minimize the mechanical impact of external stress. Due to the different mechanical properties of the corresponding subcells, narrow‐bandgap perovskites exhibit superior tensile strength compared with CIGS and Si [147, 148, 149]; therefore, all perovskite tandem architectures are expected to deliver enhanced mechanical stability against bending, as shown in Figure 6c. Similarly, incorporating organic photovoltaic materials is an effective strategy to boost the mechanical performance of perovskite‐based hybrid [150] and tandem [151] devices, as illustrated in Figure 6d. This approach not only enhances light utilization but also provides additional stress dissipation for the perovskite layer, enabling exceptional bending performance in various bending directions. Moreover, the flexible encapsulation materials (like S‐GA [152] as shown in Figure 6e) can effectively weaken the stress in the perovskite layer, [153] ensuring excellent mechanical stability under bending conditions. This also significantly mitigates issues such as lead leakage, thereby advancing the practical application of f‐PSCs [154].

## 6 Revolutionary Applications of Integrated Flexible Solar Systems

**FIGURE 7:**
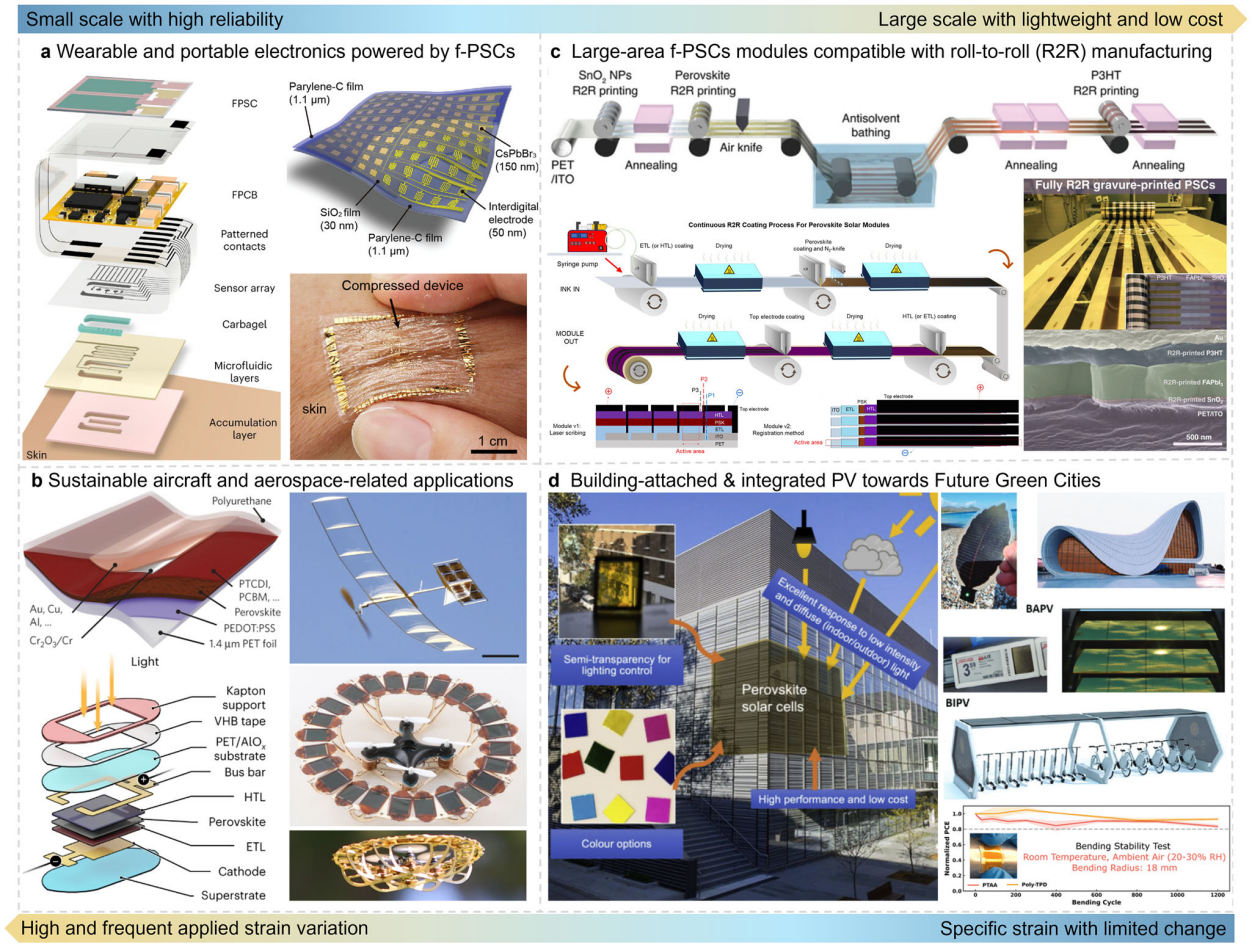
Revolutionary applications of flexible perovskite solar cells under various scenarios. a) Wearable and portable electronics powered by f‐PSCs. Reproduced with permission [156]. Copyright 2023, Springer Nature. Reproduced with permission [155]. Copyright 2021, Wiley‐VCH. b) Sustainable aircraft and aerospace‐related applications. Reproduced with permission [10]. Copyright 2024, Springer Nature. Reproduced with permission [1]. Copyright 2015, Springer Nature. c) Large‐area f‐PSCs modules compatible with R2R manufacturing. Reproduced with permission [158]. Copyright 2024, Springer Nature. Reproduced with permission [161]. Copyright 2022, American Chemical Society. d) BAPV & BIPV applications of f‐PSCs. Reproduced with permission [160]. Copyright 2022, Elsevier. Reproduced with permission [38] Copyright 2025, Wiley‐VCH.

The advancement of future green cities, the Internet of Things (IoT), intelligent manufacturing, and lifestyle innovations relies significantly on building renewable‐energy networks that are reliable, cost‐efficient, and broadly deployable [30, 31]. Benefiting from multiscale energetic mechanical understanding of f‐PSCs, flexible solar systems are poised to enable disruptive application scenarios, including four sections, as summarized in Figure 7.

For wearable and portable electronics [155] as shown in Figure 7a, small active‐area devices place the highest priority on high operational reliability, user comfort, and stable output under mixed indoor/outdoor illumination [3, 36, 150, 156]. Because these systems experience frequent bending, twisting, and intermittent strain, practical deployment further requires robust tolerance to temperature and humidity fluctuations and safe, consistent performance during daily motion [157].

In sustainable aircraft and aerospace‐related applications (Figure 7b), the key advantage of f‐PSCs is their exceptional specific power (power‐to‐weight ratio) and conformability to curved, lightweight platforms [1, 10]. Here, devices must combine high outdoor stability with mechanical robustness under complex and often time‐varying strains (e.g., vibration, aerodynamic loading, and repeated deformation), enabling extended‐duration missions while reducing payload and reliance on heavy energy storage.

For large‐area flexible modules compatible with roll‐to‐roll (R2R) manufacturing, [158] scaling up shifts the emphasis from small‐area performance to lightweight construction and low cost together with uniformity and yield, as depicted in Figure 7c [17, 38, 44, 71, 159]. Achieving mechanically durable, defect‐tolerant films and interfaces over large areas, while maintaining high‐throughput, low‐temperature processing, is central to translating laboratory advances into manufacturable module technologies.

In the fields of BAPV and BIPV, [160] the unique attributes of flexible perovskite modules, including their low carbon footprint, low levelized cost of energy (LCOE), and excellent power‐to‐weight ratio, offer architects and builders exceptional opportunities to design and construct environmentally sustainable, visually appealing, and highly livable smart building clusters [5, 17, 21]. Since mechanical loading is typically less dynamic than in wearables or aircraft, priorities shift toward long‐term outdoor durability, performance under variable irradiance, color and appearance tunability (e.g., for windows), and stable operation of large‐area systems.

## 7 Conclusion and Outlook

Beyond summarizing recent progress, this perspective advances an energy‐dissipation–centric, multiscale design logic for mechanically robust f‐PSCs. Specifically, by integrating material design and the structure property relationship at the microscale to understand the energy mechanisms in the perovskite bulk. The energy dissipation mechanism at the mesoscale highlights crosslinking and polymerized materials as a particularly powerful and tunable route to engineer dissipative networks and strengthen interfacial adhesion mitigating the crack propagation. At the macroscale, these mechanistic insights are translated into device‐structure guidelines (e.g., neutral‐plane and multilayer stress redistribution), especially for the flexible perovskite‐based tandem solar cells, thereby bridging mesoscale failure modes to macroscopic fatigue behavior and PCE retention through energetic metrics such as *w*_D_, *G*_c_, and *K*_Ic_. Although the current development of f‐PSCs is still constrained by the lack of interface transport materials with both excellent optoelectronic and mechanical properties, we believe the proposed framework provides actionable criteria and optimization pathways for future molecular design and device engineering.

Looking forward, breakthroughs in f‐PSCs are expected along several directions: i) reliable perovskite compositions and heterostructures with improved phase and crystal stability for f‐PSCs; ii) cost‐effective, high‐performance cross‐linking and polymer‐based additives and interfacial transport materials designed with mechanical energy dissipation and adhesion regulation; iii) highly tensile flexible perovskite‐based tandem devices with optical and mechanically matched as well as robust encapsulation; and iv) roll‐to‐roll (R2R) manufacturing compatible with large‐area flexible perovskite modules, accompanied by more unified mechanical‐reliability evaluation protocols. As these challenges are addressed, f‐PSCs are poised to enable transformative applications—from smart wearables and distributed IoT power to intelligent‐city infrastructure and long‐duration self‐powered aerospace systems—thereby contributing meaningfully to next‐generation clean‐energy technologies.

## Conflicts of Interest

The authors declare no conflicts of interest.

## Data Availability

The data that support the findings of this study are available in the supplementary material of this article.
